# Assessment of graft perfusion and oxygenation for improved outcome in esophageal cancer surgery: Protocol for a single-center prospective observational study

**DOI:** 10.1097/MD.0000000000012073

**Published:** 2018-09-21

**Authors:** Elke Van Daele, Yves Van Nieuwenhove, Wim Ceelen, Christiaan Vanhove, Bart P. Braeckman, Anne Hoorens, Jurgen Van Limmen, Oswald Varin, Dirk Van de Putte, Wouter Willaert, Piet Pattyn

**Affiliations:** a Department of Gastrointestinal Surgery; b Department of Electronics and Information Systems; c Department of Aging Physiology and Molecular Evolution; d Department of Pathology; e Department of Anaesthesiology, Ghent University Hospital/Ghent University, Ghent, Belgium.

**Keywords:** anastomotic leakage, esophagectomy, indocyanine green angiography, near-infrared fluorescence imaging, oxygenation, perfusion, stomach graft

## Abstract

**Introduction:**

The main cause of anastomotic leakage (AL) is tissue hypoxia, which results from impaired perfusion of the pedicle stomach graft after esophageal reconstruction. Clinical judgment is unreliable in determining graft perfusion. Therefore, an objective, validated, and reproducible method is urgently needed. Near infrared fluorescence perfusion imaging using indocyanine green (ICG) is an emerging and promising modality. This study's objectives are to evaluate the feasibility of quantification of ICG angiography (ICGA) to assess graft perfusion and to validate ICGA by comparison with hemodynamic parameters, blood and tissue expression of hypoxia-induced markers, and tissue mitochondrial respiration rates. And, second, to evaluate its ability to predict AL in patients after minimally invasive esophagectomy (MIE).

**Methods:**

Patients (N = 70) with resectable esophageal cancer will be recruited for standard MIE. ICGA will be performed after graft creation and thoracic pull-up. Dynamic digital images will be obtained starting after intravenous bolus administration of ICG. The resulting images will be subjected to curve analysis and to compartmental analysis based on the adiabatic approximation to tissue homogeneity kinetic model. The calculated perfusion parameters will be compared to intraoperative hemodynamic data to evaluate the effects of patient hemodynamics. To verify whether graft perfusion represents tissue oxygenation, ICGA perfusion parameters will be compared with systemic and serosa lactate from the stomach graft. In addition, perfusion parameters will be compared to tissue expression of hypoxia-related markers and mitochondrial chain respiratory rate. Finally, the ability of functional, histological, and cellular perfusion and oxygenation parameters to predict AL and postoperative morbidity in general will be evaluated using the appropriate univariate and multivariate statistical analyses.

**Discussion:**

The results of this project may lead to a novel, reproducible, and minimally invasive method to objectively assess perioperative anastomotic perfusion during MIE, potentially reducing the incidence of AL and its associated severe morbidity and mortality.

**Trial registration:**

Clinicaltrials.gov registration number is NCT03587532. The study was approved by the ethical committee of the Ghent University, Belgium (B670201836427).

## 1 Introduction

### 1.1 Esophagectomy

Over the past 2 decades the incidence of adenocarcinoma of the esophagus is rapidly increasing.^[1]^ Despite extensive changes in therapeutic modalities surgical resection remains the cornerstone in therapy for curable esophageal cancer (EC). After the esophagectomy, the stomach is most commonly used to restore continuity of the upper gastro intestinal tract. However, anastomotic sites are prone to serious complications such as leakage, fistulas, bleeding, and strictures. Anastomotic leakage (AL) remains one of the main causes of postoperative morbidity and mortality in digestive surgery. The reported leakage incidence after esophagectomy ranges from 5% to up to 20%.^[2–6]^ The leak-associated mortality is 18% to 40% compared with an overall in-hospital mortality of 4% to 6%.^[2,7,8]^ Several risk factors for AL have been identified.^[2–11]^ The perfusion of the anastomosis is generally considered to be an important predictor for anastomotic integrity. During surgery the clinical judgment of the surgeon determines the anastomotic region's viability based on subjective parameters as color and vessel pulsation. A good noninvasive method to objectively assess viability and perfusion of the anastomotic site could help the surgeon to reliable decide the appropriate anastomotic region.

### 1.2 Perfusion assessment methods

Several methods have been tested to intraoperatively evaluate graft's perfusion, such as Laser speckle contrast imaging, gastric tonometry, Doppler flowmetry, spectroscopy, and angiography.^[12–15]^ None of them have been widely accepted due to different reasons. Most of these methods could differentiate well-perfused from hypoperfused tissue but failed to give quantitative physiological information on vascular perfusion. Angiography is a widely used easy, intraoperative tool to provide structural information on vascularization. Quantifying angiography would combine the advantages of a structural imaging with the necessary info needed from a functional perfusion analysis.

### 1.3 NIRF imaging with ICG

Indocyanine green angiography (ICGA) is an emerging medical imaging modality requiring a fluorescence-imaging agent that can be excited at near infrared (NIR) wavelengths. Penetrating NIR light excites a near-infrared fluorescence (NIRF) agent generating fluorescence that can be captured by adapted cameras to create a 2D image of tissue deposition of the NIRF imaging agent. Indocyanine green (ICG) is a clinically used NIRF agent with excellent safety records. Its absorption maximum around 760 to 780 nm, its confinement to the vascular compartment (due to binding with plasma proteins), its low toxicity, and its rapid and almost exclusive biliary excretion has added to the rapid Food and Drug Administration approval for clinical use in 1956. ICG has been used intraoperative in neurosurgical, coronary, vascular, and reconstructive surgeries as a nonspecific blood vascular imaging agent to assess organ perfusion.^[16]^

### 1.4 ICGA-guided esophageal surgery

Objective, in-depth assessment of tissue perfusion using NIR imaging during creation of the esophagogastric anastomosis in EC surgery has not been previously reported. Most studies aiming to measure intraoperative tissue perfusion during surgery have used invasive probes or methods that sample only a small tissue volume. Several pilot studies were published that report promising results of NIRF-based perfusion imaging during EC surgery.^[17–20]^ However, these were feasibility studies, and did not encompass any attempts to calculate physiologically relevant parameters, quantify perfusion, or validate imaging findings by comparison with a criterion standard.^[17–24]^

## 2 Methods and analysis

### 2.1 Study design

This is a prospective, single-center, in vivo, observational study on 70 patients undergoing minimally invasive esophagectomy (MIE) with a pedicle gastric graft reconstruction in the department of digestive surgery at the University Hospital of Ghent. ICGA images perfusion measurements, blood samples, and biopsies will be obtained after creation of the gastric tube and before the esophagogastric anastomosis. Dynamic ICGA digital images will be obtained and subjected to curve analysis and to compartmental analysis based on the adiabatic approximation to tissue homogeneity (AATH) kinetic model. The calculated imaging based perfusion assessment of the stomach graft will be compared to intraoperative hemodynamic data and validated using tissue, serum, and cellular hallmarks of hypoperfusion and hypoxia. Finally, the ability of functional, histological, and cellular perfusion and oxygenation parameters to predict AL and postoperative morbidity in general will be evaluated using the appropriate univariate and multivariate statistical analyses.

The methodology was developed according to the strengthening the reporting of observational studies in epidemiology (STROBE) and the statement for reporting studies of diagnostic accuracy (STARD) guidelines. The protocol was written based on the Standard protocol items for clinical trials (SPIRIT) checklist.

### 2.2 Study objectives and endpoints

The primary endpoint will be to determine an ICGA-based cutoff point to predict AL and graft necrosis after esophageal reconstructive surgery. This cutoff value will be an ICGA fluorescent intensity time measurement expressed in seconds.

Secondary endpoints are the following:

- The evaluation of ICGA as a quantitative perfusion imaging modality during gastric tube reconstruction. First, intensity over time curves will be analyzed in the regions of interest to generate quantitative values for maximal fluorescence intensity (*I*_max_), inflow time (*T*_inflow_), and outflow time (*T*_outflow_). Second, a kinetic modeling analysis of the time-varying ICG concentration images will be performed, based on a tracer kinetic model that was developed to measure tissue blood flow and vascular permeability.
- The validation of ICGA imaging by comparing the quantitative data:

- To continuous perioperative hemodynamic measurements from a Pulse index Continuous Cardiac Output (PiCCO) catheter.
- To biological markers of hypoxia and ischemia: systemic and capillary lactate levels (measured in systemic and serosal blood samples) and mitochondrial chain respiratory rates (measured on tissue biopsies).
- And to histopathological ischemic markers of hypoxia and ischemia.
- Patient outcome in terms of AL and necrosis defined by the Esophagectomy Complications Consensus Group (ECCG) classification and identified by clinical and radiological methods. Based on a receiver operating curve we hope to determine a cut-off value for the prediction of gastric graft malperfusion.
- Product-related toxicity.
- Surgery-related endpoints: number of crossover patients: perioperative change of anastomotic site based on ICG findings, perioperative blood loss, and perioperative adverse events.
- Incidence of minor and major adverse events up to 30 days postoperatively classified by the Clavien-Dindo score and based on the ECCG international consensus for complications associated with esophagectomy.^[25]^
- Intensive care unit and in hospital stay and finally incidence of stricture up to 1 year postoperatively.

### 2.3 Study population

All nonmetastatic EC patients aged ≥18 years and scheduled for elective MIE with an intrathoracic stapled esophagogastric anastomosis will be screened for inclusion and exclusion criteria. Once potentially eligible, study procedures will be explained to the candidate and participation will be offered. Subjects willing to participate must be physically and mentally fit for surgery, and need to sign an informed consent after a consent process and before any study-related procedures may take place. Surgery must be within 90 days of signing the informed consent. Patients will be excluded in case of known hypersensitivity to ICG. Female patients who are pregnant or nursing, patients participating in other studies involving investigational drugs or devices, and patients with documented use of Avastin (bevacizumab) or other anti-vascular endothelial growth factor agents within 30 days before surgery. During surgery exclusion criteria will be reanalyzed and patients will be excluded in case of intraoperative findings that may preclude conduct of the study procedures, when anastomosis is performed differently than the standard of care and in case of excessive bleeding (>500 mL) before anastomosis.

### 2.4 Sample size

For the sample size we focused on the accuracy with which the ICGA quantitative parameters will be able to predict AL. For an alpha = 0.05 and a power of 0.80, a total sample size of 54 patients is required to be able to show a statistically significant difference of the area under the curve in a receiver operating characteristic curve (ROC), provided that a leak rate of 16% is observed. Including a 10% drop out or lost to follow-up, we intent to include 70 patients.

### 2.5 Study interventions

#### 2.5.1 Surgical procedure

**Figure 1:**
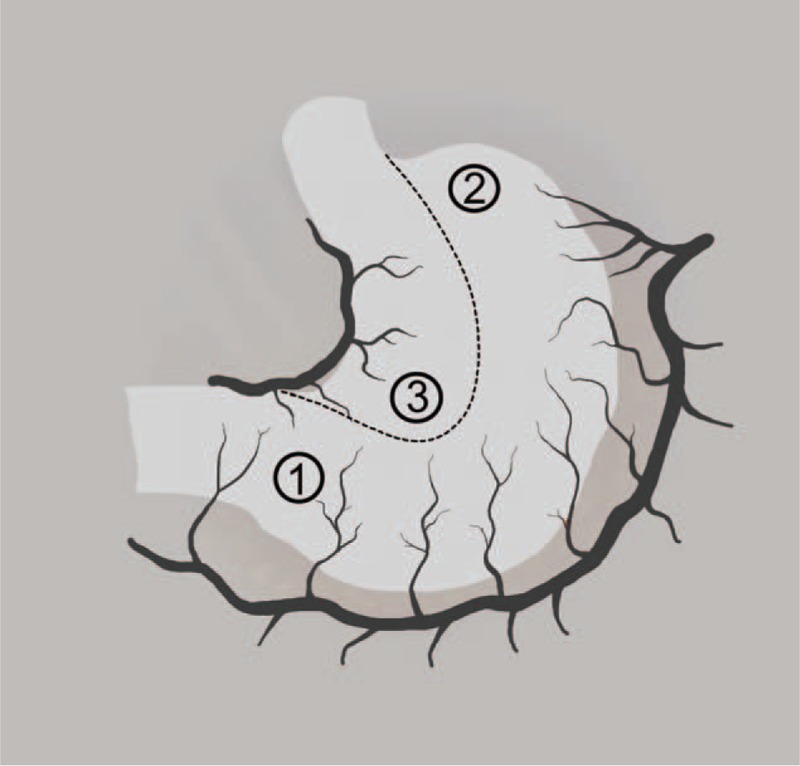
The regions of interest during gastric tube construction.

A standard MIE will be performed using the Ivor Lewis technique (intrathoracic stapled esophagogastric anastomosis). Three regions of interest (ROIs) will be marked on the stomach graft with a suture (Fig. 1):

ROI 1: well perfused control region: 2 to 3 cm proximal to the pylorus

ROI 2: clinically identified anastomotic site: study region

ROI 3: ischemic control region: in the proximal (excluded) stomach

#### 2.5.2 ICG-based NIR perfusion imaging

**Figure 2:**
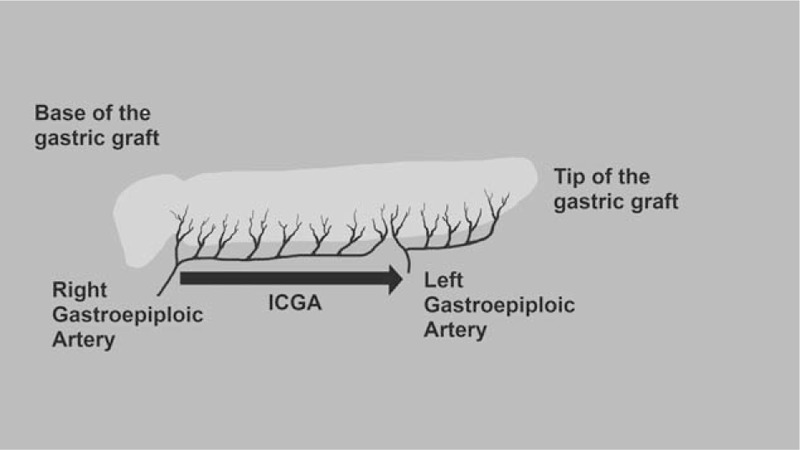
Expected ICGA perfusion through the gastric tube. ICGA = indocyanine green angiography.

Imaging will be performed twice: 30 minutes after the creation of the stomach graft (in the abdomen), and immediately before creation of the esophagogastric anastomosis (in the right thorax). A dose of 0.5 mg/kg of ICG (ICG-Pulsion, Pulsion Medical Systems, Munich, Germany) will be injected as an intravenous bolus. Images will be recorded starting immediately before injection until 3 minutes afterwards. Video data will be obtained with a charge-coupled device camera fitted with a light-emitting diode emitting at a wavelength of 760 nm, and the appropriate filters to detect NIR fluorescence (Visera elite II, Olympus Medical System Corp, Tokyo, Japan). The fluorescence signals are sent to a digital video processor to be displayed on a TV monitor in real time (Fig. 2).

#### 2.5.3 Hemodynamic evaluation

Advanced continuous hemodynamic monitoring during surgery will be performed using a PiCCO (Pulsion Medical Systems) catheter integrating a wide array of both static and dynamic data through a combination of transcardiopulmonary thermodilution and pulse contour analysis. All patients will be monitored through a central venous line in the right or left jugular or subclavian vein and a Pulsioncath thermodilution catheter in left or right femoral artery.

#### 2.5.4 Biological and pathological markers of ischemia

Blood and tissue samples will be collected at the 3 ROIs immediately after the 2 defined moments of ICGA. In order not to harm the conduit, no extra tissue will be resected compared to the standard Ivor Lewis procedure. Pathological specimen and tissue samples will be analyzed in the Department of Pathology at the University of Ghent. Biological specimens will be analyzed at the surgical research lab of the University of Ghent. Samples will be fully identified and stored at the research laboratory until the end of the study, thereafter they will be destroyed.

*The first biological marker is systemic and local capillary lactate:* systemic lactate will be measured on a blood gas sample during surgery at the beginning of the surgical procedure and at the 2 defined imaging moments. Tissue capillary lactate will be measured with a handheld lactate analyzer (EDGE Analyzer, Apex Biotechnology, Taiwan) at the same moments on blood samples obtained by a serosa puncture at the 3 ROIs.^[23,24,26]^

*The second marker of ischemia is the mitochondrial respiratory chain assessment*: Mitochondrial activity will be measured on full-thickness stomach biopsies at the previously marked ROIs. Samples will be placed in a 2-mL water jacketed oxygraphic cell equipped with a Clark type electrode (Oxygraph-2k, Ororboros Instruments, Innsbruck, Austria). Basal oxygen consumption and maximal tissue respiration rates at 37°C in the presence of a saturating amount of adenosine diphosphate as phosphate acceptor will be measured.^[23,24,26]^

*Finally, pathological analyses* will be performed on the biopsies at the 3 ROIs after they will be buffered in formalin and stained with hematoxylin and eosin. A semiquantitative scoring based on the presence of fibroblasts, polynuclear neutrophils, lymphocytes, and macrophages will be used to evaluate the severity of the inflammation. Further planned analyses (in addition to standard histology: TNM 7, Mandard regression score) include fluorescence in situ hybridization of the *MDM2* gene and immunohistochemistry analyses of ischemia-associated proteins HIF-1α, MDM2, CDK4, and p16.^[23,24,26–30]^

### 2.6 Follow-up and data collection and management

#### 2.6.1 Follow-up

Primary follow-up will be up to 30 days with clinical evaluations daily and radiological evaluations based on the standard of care of the department, and outpatient clinic follow-up visits at 30 to 60 days postoperatively. Extended follow-up will be foreseen until 1 year postoperatively for stricture.

#### 2.6.2 Data collection

All data will be retrieved from the electronic medical database and collected on Clinical Research File sheets. Three-minute long video files of the ICGA will be stored on a computer and analyzed with AMIDE ROI software (http://amide.sourceforge.net).

#### 2.6.3 Image analysis

Image analysis will be performed using curve analysis and compartmental modeling. First, intensity over time curves will be analyzed in the ROIs to generate quantitative values for maximal fluorescence intensity (*I*_max_), inflow time (*T*_inflow_), and outflow time (*T*_outflow_). Second, a kinetic modeling analysis of the time-varying ICG concentration images will be performed. This analysis will be based on a tracer kinetic model that was developed to measure tissue blood flow and vascular permeability and that has been used previously to assess vascular parameters in a tumor mode and in the brain.^[31,32]^ This complex model (AATH) will be applied on the extracted maps of perfusion and vascular permeability from the video frame rate images acquired after a bolus injection of ICG. The arterial input function of ICG, which is required for modeling, will be extracted from the images by identifying the gastric artery. This analytic work will be done in collaboration with the Imaging Division, Lawson Health Research Institute, London, Ontario.

#### 2.6.4 Data analysis

The quantitative imaging data will then be correlated with chemical and pathological markers of ischemia (Capillary lactate levels, mitochondrial chain respiratory rates, histopathological assessment, and immunohistochemical markers of ischemia) and with postoperative morbidity and mortality, including radiological or clinical AL (recorded and scored using the Clavien-Dindo scale). Based on a receiver operating curve we hope to determine a cut-off value for the prediction of gastric graft malperfusion, based on the Youden index.

### 2.7 Statistical analysis

Study data will be collected in an excel data collection sheet, in which descriptive statistics can be provided for all measured parameters. Quantitative data are expressed as mean and/or median with standard deviation, interquartile range, or min-max range. Data will be analyzed using standard statistical software (SPSS version 22 for windows, SPSS Inc, Chicago, IL). An ROC curve for sensitivity and specificity for leakage as binary classifier will be constructed. From this curve, the Youden index will be used to calculate a best-fit cut-off point. Statistical analysis using chi-square test, Fisher exact test, and Mann-Whitney *U* test will be performed whenever needed. A *P* value <0.05 is considered statistically significant.

### 2.8 Ethics

This study will be conducted in accordance with the declaration of Helsinki and in agreement with the guidelines and principles of Good clinical practice. All patients will receive oral and written information before obtaining written consent in case of approval. This final version of the protocol was approved on 12/6/2018 by the medical ethical committee of Ghent University Hospital (EC/2018/0671), registered by the Belgian research committee (B670201836427), and submitted to the clinicaltrials.gov database (NCT03587532). Enrolment will start on September 1, 2018.

## 3 Discussion

Esophageal anastomoses are fragile and prone to leakage, which is associated with significant morbidity, mortality, and impaired quality of life. The perfusion of the anastomosis is generally considered to be an important predictor for anastomotic integrity. During surgery the clinical judgment of the surgeon determines the anastomotic region based on subjective parameters. A good noninvasive method to objectively assess viability and both macro- and microvascular perfusion of the anastomotic site could help the surgeon to reliably decide the appropriate anastomotic region. Such a test needs to be easily available, cost effective, objective, and reproducible. NIRF imaging with ICG might combine all these advantages but is not yet calibrated nor validated. The proposed clinical study would be the first to use NIRF dynamic images to calculate physiologically relevant parameters (blood flow, blood volume, vascular leakage) and generate pseudocolor coded parametric maps using advanced curve analysis and compartmental modeling (AATH model).^[26,31]^ In addition, this study would be the first to validate imaging-based perfusion assessment of the stomach graft using tissue, serum, and cellular hallmarks of hypoperfusion and hypoxia. Data from this study will result in quantification of ICGA images, evaluate its potential to assess graft perfusion, and analyze its impact on AL after MIE for EC. The results of this project may allow to predict, and therefore avoid, AL after EC surgery. This may, in turn, prevent the morbidity and mortality associated with AL. Also, the results might contribute to a better understanding of gastric graft perfusion and oxygen supply in EC surgery.

In conclusion, this study will produce quantitative perfusion-related measurements for an objective ICGA assessment of tissue perfusion during surgery. The insights gained and the methods developed during this project may also benefit patients undergoing other types of cancer surgery (rectum, pancreas…) in which anastomotic tissue viability is of critical importance.

## Acknowledgments

The authors would like to express their gratitude to Inge Van den Broucke, Patricia Horckmans, Leen Degezelle, and Evelien Dierick for their continuous support and help in the development and fulfillment of this study. Moreover, we would like to thank “Kom op tegen Kanker” for their continuous financial support in cancer research.

## Author contributions

**Conceptualization:** Elke Van Daele, Yves Van Nieuwenhove, Wim Ceelen, Piet Pattyn.

**Data curation:** Elke Van Daele, Christiaan Vanhove, Bart Braeckman, Anne Hoorens, Jurgen Van Limmen, Piet Pattyn.

**Formal analysis:** Oswald Varin.

**Funding acquisition:** Elke Van Daele, Yves Van Nieuwenhove, Wim Ceelen, Piet Pattyn.

**Investigation:** Elke Van Daele.

**Methodology:** Elke Van Daele, Yves Van Nieuwenhove, Wim Ceelen, Christiaan Vanhove, Bart Braeckman, Anne Hoorens, Jurgen Van Limmen, Oswald Varin, Piet Pattyn.

**Project administration:** Elke Van Daele.

**Resources:** Piet Pattyn.

**Software:** Christiaan Vanhove.

**Supervision:** Yves Van Nieuwenhove, Oswald Varin, Piet Pattyn.

**Validation:** Elke Van Daele.

**Visualization:** Christiaan Vanhove.

**Writing – original draft:** Elke Van Daele.

**Writing – review and editing:** Yves Van Nieuwenhove, Wim Ceelen, Jurgen Van Limmen, Dirk Van de Putte, Wouter Willaert.
